# A mutation in the interferon regulatory element of HBV may influence the response of interferon treatment in chronic hepatitis B patients

**DOI:** 10.1186/1743-422X-9-10

**Published:** 2012-01-10

**Authors:** Jia-Jie Lu, En-Qiang Chen, Jia-Hong Yang, Tao-You Zhou, Li Liu, Hong Tang

**Affiliations:** 1 Center of Infectious Diseases, West China Hospital, Sichuan University, Chengdu, China; 2 Division of Infectious Diseases, State Key Laboratory of Biotherapy, Sichuan University, Chengdu, China; 3 Department of Infectious diseases, People's Hospital of Deyang City, Deyang City, China

## Abstract

**Background:**

A functional interferon regulatory element (IRE) has been found in the EnhI/X promoter region of hepatitis B virus (HBV) genome. The purpose of this study is to compare the gene order of responder and non-responder to interferon therapy in patients with chronic hepatitis B (CHB), so as to evaluate the relationship between IRE mutation and the response to interferon treatment for CHB patients.

**Results:**

Synthetic therapeutic effect is divided into complete response (CR), partial response (PR) and non-response (NR). Among the 62 cases included in this study, 40 cases (64.5%) were in the response group (CR and PR) and 22 (35.5%) cases were in the NR group. Wild type sequence of HBV IRE TTTCACTTTC were found in 35 cases (56.5%), and five different IRE gene sequences. included TTTtACTTTC, TTTCAtTTTC, TTTtAtTTTC, TTTtACTTTt and cTTtACcTTC, were found in 22 cases (35.5%), 1 case (1.6%), 1 case (1.6%), 2 cases (3.2%) and 1 case (1.6%) respectively. There were 41.9%cases (26/62) with forth base C→T mutation, consisted of 32.5% (13/40) cases in response group and 59.1% (13/22) cases in NR group. Among the 35 cases with IRE sequences, there were 67.5% (27/40) cases in response group and 36.4% (8/22) in NR group, and the difference in IRE sequences between two groups was statistic significantly (P = 0.027). The result suggested that there is likely relationship between the forth base mutation (C→T) of IRE region and the response of HBV to Interferon therapy, and this mutation may partially decrease the inhibition effect of interferon on HBV.

**Conclusion:**

The forth base C→T mutation in IRE element of HBV may partially influence the response of Interferon treatment in CHB patients.

## Background

Hepatitis B virus (HBV) infection is a serious global health problem, with 2 billion people infected worldwide, and 350 million suffering from chronic HBV infection [1]. It's estimated that over 1 million HBV infected patients died annually because of hepatic decompensation, cirrhosis and hepatocellular carcinoma (HCC)[2,3]. Evidence showed that antiviral treatment is the only way to reduce morbidity and mortality from chronic HBV infection.

Currently, antiviral treatments for chronic hepatitis B (CHB) include interferon (IFN) therapy and nucleoside analogues [4]. Because of anti-HBV and immunoregulation dual functions [5-11], IFN has been widely used in past decade. However, existing clinical data suggested that the anti-viral effect of IFN is not satisfactory at present; for patients with active liver inflammatory, the efficiency of IFN is only about 30%-50% in clinical observation, and 50%-70% patients had no response. As one of the main anti-HBV drugs, the action site and mechanism of interferon are not completely clear. Evidence shows that the inhibition of virus replication by IFN needs that the cell can activate antivirus effect, and also the virus is sensitive to the action. As the sensitivities of different HBV strains (like the different genotype or some district of the gene variations) to the interferon are different [12,13]. Therefore, the anti-HBV effect of interferon is affected by the factors of both host and virus.

Recent studies indicate that the inhibition of virus replication by interferon may influence both transcriptional and post-transcriptional regulation [14,15]. The antiviral effect of interferon is through the activation of JAK-STAT signal transduction pathways. Interferon induces the formation of a heterotrimetric transcription factor complex, interferon-stimulated gene factor 3 (ISGF3), which consists of signal transducers and activators of transcription 1 and 2 (STAT1 and STAT2) and interferon regulate factor 9(IRF9), after binding to its receptor. ISGF3 translocates into the nucleus, binds to the interferon-stimulated regulatory element (ISRE) and transactivates the expression of Interferon stimulated genes (ISG) such as the Interferon regulatory factor 7 (IRF7), the Interferon regulatory factor 1 (IRF1) and the Interferon regulatory factor 2 (IRF2) etc. There is a functional interferon regulatory element (IRE) in HBV enhancerI/X promoter region [16], which can mediated the regulation of gene transcription by IFN α, the Interferon-stimulated 3 (ISGF3), the Interferon regulatory factor 1 (IRF1) and the Interferon regulatory factor 7 (IRF7)[17]. It was suggested that interferon probably regulates HBV gene transcription directly through the IRE sequence. However, the role of IRE in the effect of interferon in CHB treatment is still not clear. In this study, the sequences of IRE region of HBV from responder and non- responder to interferon therapy in patients with CHB were compared, and the relationship between IRE mutation and the response to interferon were analyzed.

## Results

### General information

Among those 62 patients, thirty four males and six females were in the response group, aged from 3 to 51 years (28.9 ± 11.0 years), and seventeen males and five females were in the non-response group, aged from 16 to 45 years (28.0 ± 8.3 years). Fifty-three patients were positive for HBeAg and nine patients were negative for HBeAg (Table 1).

**Table 1:** Baseline characteristics and disease status of patients

	Response	Non-response
Patients, n	40	22
Age, years(mean ± SD)	28.9 ± 11.0	28.0 ± 8.3
Sex, male(no.,%)	34(85.0)	17(77.3)
Pathogenesis, days(mean ± SD)	1719.5 ± 1168.0	1503.8 ± 1322.1
Family history(no.,%)	7(17.5)	2(9.0)
HBV DNA, log copies/ml(mean ± SD)	5.1 ± 0.9	5.4 ± 1.0
ALT, IU/L(mean ± SD)	260.9 ± 84.0	248.1 ± 112.0

### Response to the Interferon treatment

As to serum virological response (SVR) there were 20 cases for complete response group, in which 16 cases were positive for HBeAg while 4 cases were negative for HBeAg. There were 18 cases for partial response group, in which 15 cases were positive for HBeAg and 3 cases were negative for HBeAg. There were 24 cases for non-response group, in which 22 cases were positive for HBeAg and 2 cases were negative for HBeAg.

As to SIR there were 16, 7 and 30 cases each for complete, partial and non-response group. In addition as to HBsAg there was one case each in HBeAg-positive and HBeAg-negative group turned negative, with appearance of anti-HBs in the former group and without it in the latter group at the end of six months treatment.

There were 35 cases with ALT recovered in which 2 cases (5.7%) were in SVR non-response group, 15 cases (42.9%) in SVR partial response group and 18 cases (51.4%) in SVR complete response group. There were 27 cases with ALT failed to return to normal, in which 22 cases (81.5%) were in SVR non-response group, 3 cases (11.1%) in SVR partial response group and 2 cases (7.4%) in SVR complete response group.

Combined the results of SVR, SIR and SBR together, the therapeutic effect evaluation results were as following: 13 cases were as CR; 27 cases were as PR and 22 cases were as NR. Therefore 40 cases were in response group and 22 cases were in non-response group.

### Different patterns of mutation in the IRE region of HBV

**Figure 1:**
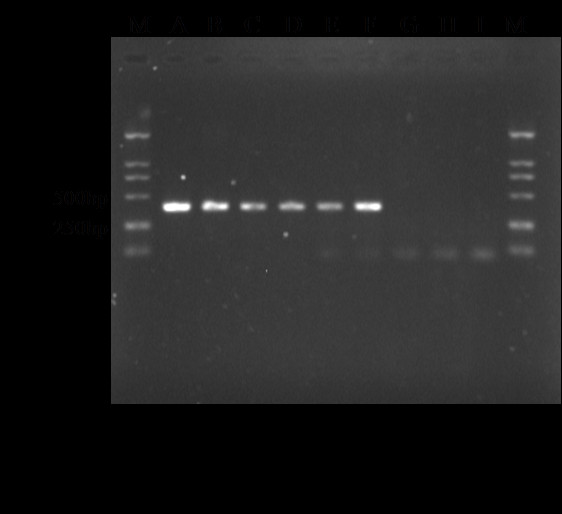
**PCR products of HBV DNA including IRE region from the serum of patients and control**. M: DNA marker DL2000 A: pHBV4.1 plasmid B: pBHB 4 plasmid C: Chronic hepatitis B patient's serum(positive control) D-F:our patient's serums G:PAd/NS3 plasmid H: Serum of normal person I: TE (blank control).

**Figure 2:**
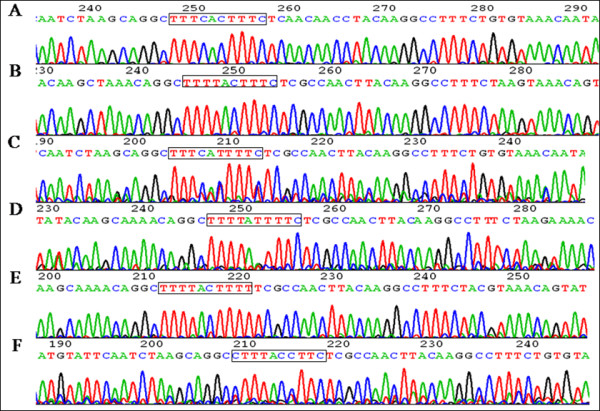
**IRE sequences of wild strains and mutation strains**. A: wild strain B: forth base point mutation(C→T) C: sixth base point mutation(C→T) D: forth base point mutation(C→T) and sixth base point mutation(C→T) E: forth base point mutation(C→T) and tenth base point mutation(C→T) F: first base point mutation(T→C), forth base point mutation(C→T) and seventh base point mutation(T→C).

As shown in Figure 1, the size of target band containing IRE was 371 bp. Sequence analysis showed there were six kinds of IRE sequences variants as shown in Figure 2. The wild type sequence was TTTCACTTTC according to the sequence of 12 ayw HBV strains and 15 adr HBV strains. Among the 62 patients, there were 35cases (56.6%) with wild type IRE and 27 cases with variant IRE sequences. Five different IRE variants were TTTtACTTTC (22 cases, 35.5%), TTTCAtTTTC (1 case, 1.6%), TTTtAtTTTC (1 case, 1.6%), TTTtACTTTt (2 cases, 3.2%) and cTTtACcTTC (1 case, 1.6%).

### Comparison of HBV IRE mutation between response and non-response group

There were 27 responders and 8 non-responders with wild type IRE sequences TTTCACTTTC, while there were 10 responders and 12 non-responders with mutant IRE sequences TTTtACTTTC (Table 2).

**Table 2:** Comparison of HBV IRE gene sequences between response and non-response group

HBV IRE gene sequence	Response (n = 40)	Non-response (n = 22)	Total (n = 62)
TTTCACTTTC	27	8	35
TTTtACTTTC	10	12	22
TTTCAtTTTC	0	1	1
TTTtAtTTTC	0	1	1
TTTtACTTTt	2	0	2
cTTtACcTTC	1	0	1

There were 10 complete responders to interferon, 17 partial responders and 8 non-responders in the patients whose IRE sequences were TTTCACTTTC, while there were 3 complete responders to interferon, 7 partial responders and 12 non-responders in the patients whose IRE sequences were TTTtACTTTC.

Other four kinds of point mutation including sixth base C→T mutation(2 cases, TTTC AtTTTC, TTTtAtTTTC), tenth base C→T mutation (2 cases, TTTtACTTTt), first base T→C mutation(1 case, cTTtACcTTC) and seventh base T→C mutation(1 case, cTTtACcTTC) only account for 8% of total sample.

There were 26 cases with forth base C→T mutation (TTTtACTTTC 22 cases, TTTtAtTTTC 1 case, cTTtACcTTC 2 cases, TTTtACTTTt 1 case). Among them, 13 cases in response group and 13 cases in non-response group. There were 35 cases with wild strain IRE sequences (TTTCAC TTTC) which included 27 responder and 8 non-responders. According to the statistical analysis, the chi-square value was 4.869 and P value was 0.027(P < 0.05). The results showed that the forth base mutation (C→T) of IRE region presented more frequent in the non-response group, suggested that there's likely a relationship between the forth base mutation(C→T) of IRE region and the response to Interferon treatment.

## Methods

### Patients and treatment

Sixty two serum samples of chronic hepatitis B outpatients from West china hospital, Sichuan University and People's Hospital of Deyang City, Sichuan, China from 2004 to 2005 were included in this study. Patients eligible for the initial study had been positive for HBsAg for more than 6 months, had elevated serum alanine aminotransferase (ALT) levels of 2-10 times the upper limit of normal (ULN), and had a serum HBV DNA level ≥100,000 copies/mL (≥10,000 copies/ml for HBeAg- negative patients). Exclusion criteria were as follows: antiviral or immunosuppressive therapy within the previous 6 months; coinfection with hepatitis C, hepatitis D, or human immunodeficiency virus; other acquired or inherited causes of liver disease; and preexisting cytopenia or decompensated liver disease.

The adult patients received subcutaneous injection of 5,000,000^u ^interferon-α every other day for 6 months, and the children less than 10 years old received subcutaneous injection of 3,000,000^u ^interferon-α every other day for 6 months.

This study was approved by West China Hospital's institutional review board and was conducted per the 1975 Declaration of Helsinki. All patients signed informed consent forms before their inclusion in this study.

### Efficacy evaluation

The serum virology responses (SVR) were divided to complete response (decreasing of serum HBV DNA to less than 1000 copies/ml), partial response (the decreasing of HBV-DNA was more than 2 log_10 _IU/ml but can still be detected) and non-response (the decreasing of HBV-DNA was negligible).

The serum immunological responses (SIR) were divided to complete response (loss of HBeAg and appearance of anti-HBe in HBeAg positive patients), partial response (loss of HBeAg without anti-HBe) and non-response (HBeAg positive and anti-HBe negative).

The serum bio-chemical response (SBR) were divided to complete response (decreasing of serum ALT to normal level) and non-response (the decreasing of serum ALT was negligible).

The therapeutic effect evaluation were divided to complete response (CR, SVR, SIR and SBR were all complete response at the end of treatment), non-response (NR, SVR, SIR and SBR were all non-response) and partial response (PR, all of the rest of mentioned). The patients were divided into response group (including complete response and partial response) and non-response group.

### Hepatitis B virus nucleotide sequencing

According to AY721605 strain HBV whole genome sequence, primers for HBV IRE sequence amplification were designed (Sense: GCGCG GTACC GGGTA TACAT TTAAA CCCTA; Antisense: TTAAA GCTTG CGTCA GCAAA CA CTT GGC). And the primers were synthesized by Invitrogen China.

After viral DNA isolation PCR was carried out to amplify IRE sequences. Briefly the reaction was done in a 50 μL volume. The PCR conditions were: initial heating at 94°C (5 minutes), denaturing at 94°C (45 seconds), annealing at 55°C (45 seconds), and extension at 72°C (45 seconds). A total of 35 cycles were performed, followed by a final extension at 72°C (5 minutes). The PCR products were sequenced by Invitrogen China.

### Statistical analysis

All results were expressed as median and standard deviation (*X *± s). All data were analyzed using SPSS 11.5. A chi-square test was used to determine whether there was a difference between the groups. P-value of < 0.05 was considered statistically significant.

## Discussion

The pathogenesis of CHB shows that persistent replication of HBV is closely related to the disease process. So using effective drugs in time to inhibit HBV amplification is very important to interrupt the disease process. Interferon has been considered effective while treating CHB. It was effective only in some patients, but ineffective when treating other patients. In this study, as to SVR in the 62 patients who received standard IFN treatment for six months, the complete response rate was 21.0% (13/62), partial response rate was 43.5% (27/62) and non-response rate was 35.5% (22/62). After a six-month treatment, as to SIR the rate of HBeAg, HBV DNA and HBsAg loss was 43.4% (23/53), 30.2%(16/53), 1.9%(1/53) respectively in HBeAg positive patients. The rate of HBV DNA and HBsAg loss was 44.4% (4/9), 11.1% (1/9) respectively in HBeAg negative patients. The rates in present study were higher as compared to some other clinical studies [10], which may correlated to the definition of therapeutic effect evaluation (complete response, partial response and non-response). Additionally, limited samples, short course of treatment and short follow-up survey also may be correlated to this high response rates.

Anti-HBV effect of IFN is thought to act through JAK-STAT signaling pathway [18,19]. In this pathway STAT1, STAT2 and IRF9 formed trimer complex called the gene factor of the Interferon-stimulated 3 (ISGF3). The ISGF3 combines with the Interferon- stimulated response element (ISRE) sequence after transporting to the cell nucleus, then activates the expression of Interferon Stimulated Genes (ISG) such as the Interferon Regulatory Factor 7 (IRF7), the Interferon Regulatory Factor 1 (IRF1) and the Interferon Regulatory Factor 2 (IRF2) etc, thus inhibit viral replication and gene expression. Therefore, the anti-HBV effects of IFN are affected by both the host and virus. According to current knowledge, the host factors may include the degree of liver inflammation, the level of transaminase, IL-12 variation during IFN therapy, the basic status of DCs, the expression of α/β receptor of the PBMCs and the levels of HLA-DRB1 DT before and after the therapy. The virus factors include the initiation level of HBV DNA, the decrease amplitude after one month treatment, genotypes and the mutations of DNA [20].

Recently, several studies showed that HBV gene mutation may affect IFN therapy [17,21]. A study with 48 HBeAg positive CHB patients who received IFN treatment for more than 24 weeks showed that the patients with nt1762 and nt1764 mutation had a poorer response to IFN with lower HBV DNA decrease and HBeAg conversion [22]. In addition the loss of HBeAg was related not only to nt1762 and nt1764 mutation (69.5%) but also closely to nt1896 (100%) and nt1814 mutation (65.2%). When these mutations occurred the antiviral efficacy of IFN was not ideal and the other antiviral strategies should be adopted. Another study showed that the T1762-A1764 mutation of HBV DNA might affect the clearance of the virus [20]. It suggested that virus with T1762- A1764 mutation had strong response to IFN therapy. IFN was not only able to clear HBV directly but also can eliminate HBV by activating or strengthening immune system.

It has been reported that there was a functional IRE in HBV enhancerI/X promoter region [16], which can mediate the regulation of gene transcription by IFN α, gene factor of the IFN-stimulated 3, IFN regulatory factor 1 (IRF1) and IFN regulatory factor 7 (IRF7)[17]. It suggested that IFN probably regulated HBV gene transcription directly by IRE. Therefore it is possible that the mutation of IRE may partially block or reduce the inhibition of IFN to HBV, causing no response occurring. In this study we found that there were six kinds of HBV IRE sequences TTTCACTTTC, TTTtACTTTC, TTTCAtTTTC, TTTtAtTTTC, TTTtACTTTt and cTTtACcTTC in the CHB patents included in our study. Comparing sequence of IRE of wild strains, we found that there were 35 cases (56.6%) among 62 patients in our study. There were five kinds of point mutation sequences. Except for the forth base C→T mutation, the other four kinds of point mutation, including the sixth base C→T mutation (2 cases), the tenth base C→T mutation (2 cases), the first base T→C mutation(1 case) and the seventh base T→C mutation(1 case), only accounted for 8% of total sample. These events may relate to random mutation or errors occurring during transcription.

In summary, the forth base C→T mutation in IRE element of HBV may partially influence the response of Interferon treatment in CHB patients. We will carry on in vitro test in order to confirm whether the forth base C→T mutation was specific mutation related to the anti-HBV effect of interferon. In addition, we still collect serum specimen of patients with no response to interferon in the hope of confirm the correlation between the HBV IRE mutation and no response to interferon with larger sample size.

## Competing interests

The contents are solely the responsibility of the authors and do not necessarily represent the views of the funding source.

## Authors' contributions

TH conceived the study, provided fund supporting and revised the manuscript critically for important intellectual content. LJJ made substantial contributions to specimen collection, analysis and interpretation of data. CEQ, YJH, ZTY, and LL participated in interpretation of data and manuscript preparation.

All authors have read and approved the final manuscript.
